# Response: New neurons from old beliefs in the adult piriform cortex? A Commentary on: “Occurrence of new neurons in the piriform cortex”

**DOI:** 10.3389/fnana.2015.00079

**Published:** 2015-06-09

**Authors:** Ti-Fei Yuan, Yu-Xiang Liang, Kwok-Fai So

**Affiliations:** ^1^School of Psychology, Nanjing Normal UniversityNanjing, China; ^2^State Key Laboratory of Brain and Cognitive Sciences, the University of Hong KongHong Kong, China; ^3^Department of Anatomy, Li Ka Shing Faculty of Medicine, The University of Hong KongHong Kong, China; ^4^GHM Institute of CNS Regeneration, Jinan UniversityGuangzhou, China; ^5^Department of Ophthalmology, Li Ka Shing Faculty of Medicine, The University of Hong KongChina

**Keywords:** adult neurogenesis, piriform cortex, epilepsy, neurodegeneration, Alzheimer's disease, Parkinson's disease

In the commentary by Nacher and Bonfanti (2015) on our recent paper in Frontiers of Neuroanatomy (Yuan et al., 2015), they argued that subpopulations of layer II cells in mammalian paleocortex, including the piriform cortex (PC), are embryonic origin-immature neurons expressing “plastic” neuronal markers, and only a limited number of PC neurons are adult generated. We thank these two experts for their comments, and acknowledge the fact that evidence for “adult neurogenesis in PC” is still limited.

We, however, wish to clarify several points as below.

First, we did not use the phrase “adult neurogenesis in PC” in the title, since the reviewer had already pointed out that it might be misleading. Hence, our paper is ultimately named “Occurrence of new neurons in the PC.” This title acknowledges that in adult PC, the full evidence of neurogenesis (proliferation, migration, differentiation, integration, functioning) is yet incomplete.

Second, Nacher and Bonfanti mentioned that pulse-labeling of BrdU revealed a low number of new neurons in adult PC, thereby questioning that adult neurogenesis can contribute to the presence of numerous cells expressing immature neuronal markers. Meanwhile, they also mentioned that these neurons could be embryonically produced. Although their statement potentially provides an alternative explanation for the abundant “new neurons” in PC, it is noteworthy that limitations of BrdU labeling have been extensively discussed (Taupin, 2007); to list a few: (1) BrdU labeling, even administered using the pulse protocol, would not fully pick up those very slowly-dividing cells, especially considering that PC is not an “active” site of new neuron occurrence; (2) Any neural stem cells could reuse the BrdU molecule released from dead cells that have been embryonically BrdU-labeled, or just uptake BrdU through the intracellular transfer (Burns et al., 2006); this might somehow lead to overestimation of the significance of embryonic neurogenesis to the overall cell generation in PC; (3) The incorporation of BrdU in the embryonic period might even affect the differentiation of neural progenitor cells in adulthood (Wilt and Anderson, 1972; Lehner et al., 2011), which would cause underestimation of the role of adult neurogenesis in the overall cell generation in PC. We do hope that retroviral based lineage tracing study would finally solve these questions.

Third, in our previous Figure 1A (modified from Klempin et al., 2011; Yuan et al., 2015), we showed that the subventricular zone (SVZ) is the only origin of PC “new neurons.” As pointed out by Nacher and Bonfanti, we will be happy to include the embryonic origin as an important source for these new neurons. We have attached a revised form of Figure 1.

**Figure 1:**
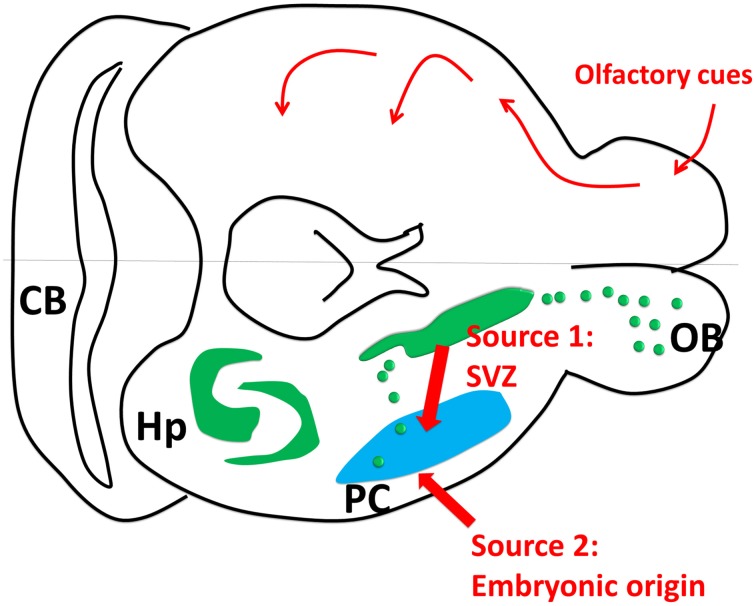
**Piriform cortex neurogenesis**. The upper part of the cartoon shows the olfactory sensory pathway. The olfactory bulb transmits the signal to the piriform cortex as the first relay, and then to other connected regions such as hypothalamus, endorhinal cortex and amygdala. The lower part of the cartoon shows the neurogenic sites including hippocampus and subventricular zone (SVZ). *Source 1:* SVZ gives birth to some new neurons in olfactory bulb and piriform cortex through rostral and caudal migration streams. *Source 2:* Some new neurons in PC are generated embryonically (Figure 1 was modified from Klempin et al., 2011).

Fourth, Nacher and Bonfanti suggested a series of nice literature talking about PSA-NCAM changes in brain aging and that PSA-NCAM might be taken as a general neuronal marker for neural plasticity rather than neurogenesis. We completely agree. However, the focus of our mini-review has been the potential of new neuron formation in piriform cortex; therefore, we cited several papers using PSA-NCAM as the new neuron marker.

Last but not least, Nacher and Bonfanti discussed that due to technical artifacts, several papers showing PC neurogenesis are needed to be reviewed cautiously. We completely agree with their view; and regard this to be the general technical difficulty in the field working on adult neurogenesis. Therefore, instead of jumping out for a full conclusion today, we prefer leaving these open possibilities in our review to the future investigations.

In summary, Nacher and Bonfanti presented a nice addition to the missing aspects in our previous mini-review. We thank their comments again; yet the potential importance and the values for future investigations of this topic are not challenged.

## Conflict of interest statement

The authors declare that the research was conducted in the absence of any commercial or financial relationships that could be construed as a potential conflict of interest.
